# Chemotherapy management of a pediatric B-ALL patient with moyamoya disease: a case report

**DOI:** 10.3389/fonc.2026.1733347

**Published:** 2026-02-19

**Authors:** Yi-ren Cheng, Juan Han, Zi-yi Huang, Ping Qu, Fen Zhou

**Affiliations:** Department of Pediatrics, Union Hospital Affiliated to Huazhong University of Science and Technology, Wuhan, Hubei, China

**Keywords:** antithrombotic strategies, B-cell acute lymphoblastic leukemia, chemotherapy management, moyamoya disease, neurological complications

## Abstract

Moyamoya disease is a rare, chronic cerebrovascular disorder characterized by progressive stenosis or occlusion of the terminal portions of the internal carotid arteries and the subsequent development of abnormal compensatory vascular networks. It represents a significant cause of intracranial arteriopathy and is a major etiology of stroke and transient ischemic attack in pediatric populations. We present the case of a 9-year-old boy with confirmed diagnosis of B-cell acute lymphoblastic leukemia (B-ALL), in whom brain MRI findings were suggestive of Moyamoya disease. This report underscores the rarity of the co-occurrence of B-ALL and Moyamoya disease and highlights the importance of early recognition and timely intervention during chemotherapy to reduce the risk of neurological complications. Our findings may offer valuable insights for the chemotherapeutic management of ALL patients with coexisting Moyamoya disease.

## 1 Introduction

Moyamoya disease is a cerebrovascular occlusive disorder of unknown etiology, characterized by stenosis or occlusion of the terminal segments of the bilateral internal carotid arteries. This vascular narrowing leads to reduced cerebral blood flow and an elevated risk of cerebral ischemia. In response to chronic hypoperfusion, a compensatory network of abnormal collateral vessels develops at the base of the brain. These vessels exhibit a characteristic “puff of smoke” appearance on cerebral angiography, from which the disease derives its name—”moyamoya” in Japanese (1, 2).

We report a case of B-cell acute lymphoblastic leukemia (B-ALL) complicated by Moyamoya disease, a condition that appears to be relatively rare in the published literature. Previous studies have suggested that Moyamoya disease in ALL patients typically arises as a long-term complication of prophylactic cranial irradiation or chemotherapeutic agents (3–5). In the present case, however, cerebral vascular stenosis was detected on neuroimaging prior to the initiation of any chemotherapy. This finding indicates that Moyamoya disease was not a treatment-related complication of B-ALL, but rather an independently coexisting condition.

Chemotherapy remains the cornerstone of treatment for B-ALL, yet it is associated with a spectrum of adverse effects, notably neurological complications. Studies indicate that approximately 3.6% to 11% of pediatric ALL patients develop acute neurological adverse events during treatment (6). The most frequently reported central nervous system toxicities include posterior reversible encephalopathy syndrome (PRES), cerebral venous sinus thrombosis, and seizures (7, 8). Among chemotherapeutic agents, asparaginase, methotrexate, and vincristine are most commonly implicated in neurotoxicity.

Given the inherent susceptibility to cerebrovascular events in Moyamoya disease and the imperative to sustain effective anti-leukemic therapy in B-ALL, optimizing chemotherapy regimens to minimize neurological complications while maintaining therapeutic efficacy poses a significant clinical challenge.

## 2 Case presentation

**Figure 1:**
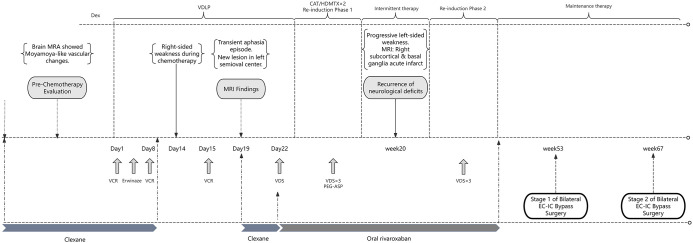
Schematic overview of the treatment course for the patient.

A 9-year-old male patient was referred to our hospital following the identification of bicytopenia in peripheral blood. Physical examination revealed no significant abnormalities. Bone marrow cytology demonstrated a blast cell proportion of 39%, and immunophenotyping confirmed the diagnosis of B-ALL. Molecular and cytogenetic analyses showed no fusion genes and yielded negative results on ALL-FISH testing. Karyotype analysis revealed a normal male chromosomal complement (46,XY). Based on the patient’s clinical features and MICM classification, a diagnosis of low-risk B-ALL was established. A schematic overview of the treatment course is provided in **Figure 1**.

**Figure 2:**
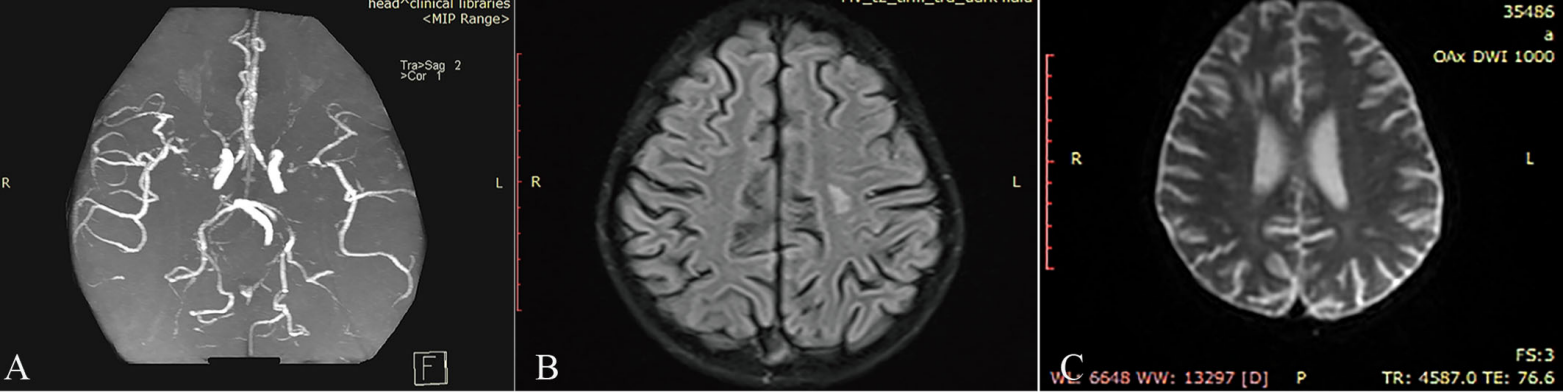
**(A)** Pre-chemotherapy brain MRA: segmental interruptions of bilateral internal carotid artery (ICA) siphon segments and middle cerebral arteries (MCA), with severe stenosis/occlusion and multiple tortuous collateral vessels. **(B)** Brain MRI: new high signal in the left semioval center on T2-weighted sequences, suggestive of ischemic lesion or demyelination. **(C)** Brain MRI DWI: new acute ischemic lesions in the right basal ganglia, indicating recent cerebral infarction.

Prior to the initiation of chemotherapy, brain magnetic resonance angiography (MRA) demonstrated segmental interruptions in the bilateral internal carotid arteries siphon segments and middle cerebral arteries, with evidence of severe stenosis or occlusion, along with the presence of multiple tortuous collateral vessels. In addition, irregular luminal diameters and intermittent visualization were noted in segments of the bilateral anterior and posterior cerebral arteries, suggestive of localized stenosis (**Figure 2A**). The patient had no history of radiotherapy or previous chemotherapy. Comprehensive follow-up evaluations had excluded infectious or autoimmune etiologies, and genetic testing for known pathogenic genes associated with Moyamoya disease (including RNF213) remained incomplete. Based on the current clinical manifestations and neuroimaging findings, the case was diagnosed as idiopathic moyamoya disease. Laboratory investigations revealed elevated levels of D-dimer and fibrin degradation products (FDP), consistent with a hypercoagulable state; accordingly, therapeutic anticoagulation with low molecular weight heparin (Clexane) was initiated. Treatment was commenced following the Chinese Children’s Cancer Group ALL 2020 (CCCG-ALL-2020) protocol. Considering the patient’s cerebrovascular abnormalities and the associated increased risk of neurological complications, pegylated asparaginase (PEG-ASP) was substituted with short-acting Erwinia asparaginase.

During treatment with the VDLP regimen, the patient developed neurological symptoms following two doses of vincristine (VCR), including right upper limb weakness, decreased dexterity of the right hand, and a limping gait. After a third VCR dose, the patient experienced transient aphasia lasting approximately 10 minutes, which resolved spontaneously. Brain MRI revealed a new abnormal signal intensity in the left semioval center, suggestive of an ischemic lesion or demyelinating change (**Figure 2B**). Clexane was initiated for anticoagulation, and dopamine was administered to enhance cerebral perfusion. A rehabilitation consultation was obtained, and early interventional rehabilitation was implemented. In light of the underlying Moyamoya-related cerebrovascular pathology and the elevated risk of chemotherapy-induced neurotoxicity, the treatment regimen was modified by substituting vincristine with vindesine (VDS), with the aim of mitigating neurotoxic risk while maintaining antileukemic efficacy.

The patient was subsequently discharged on long-term oral rivaroxaban to reduce the risk of recurrent cerebrovascular events. He successfully completed consolidation and the first re-induction cycle without further central nervous system complications. During re-induction, three doses of VDS and one dose of PEG-ASP were administered.

During the subsequent interphase treatment (week 20), the patient presented with a four-day history of progressive left-sided limb weakness, manifesting as impaired fine motor skills in the left hand and an inability to bear weight on the left lower limb. Neurological examination confirmed left-sided motor deficits, with muscle strength graded at 4 on the Medical Research Council (MRC) scale. Emergency brain MRI revealed new acute ischemic lesions in the right subcortical region and basal ganglia, indicative of recent cerebral infarction (**Figure 2C**). Treatment for dehydration and elevated intracranial pressure was initiated. In view of the patient’s borderline low platelet count, rivaroxaban was temporarily suspended and replaced with Clexane. The recurrent cerebral ischemic events following chemotherapy were considered potentially attributable to chemotherapy-induced vascular endothelial dysfunction and alterations in coagulation pathways.

Given the patient’s history of multiple Moyamoya disease-related cerebral infarctions, surgical intervention was clinically indicated. However, since the consolidation phase of leukemia treatment remained incomplete, a multidisciplinary decision was made—following detailed discussion with the family—to proceed with the second re-induction therapy according to the original protocol, while omitting asparaginase to mitigate thrombotic risk and preserve the overall intensity of anti-leukemic treatment.

**Figure 3:**
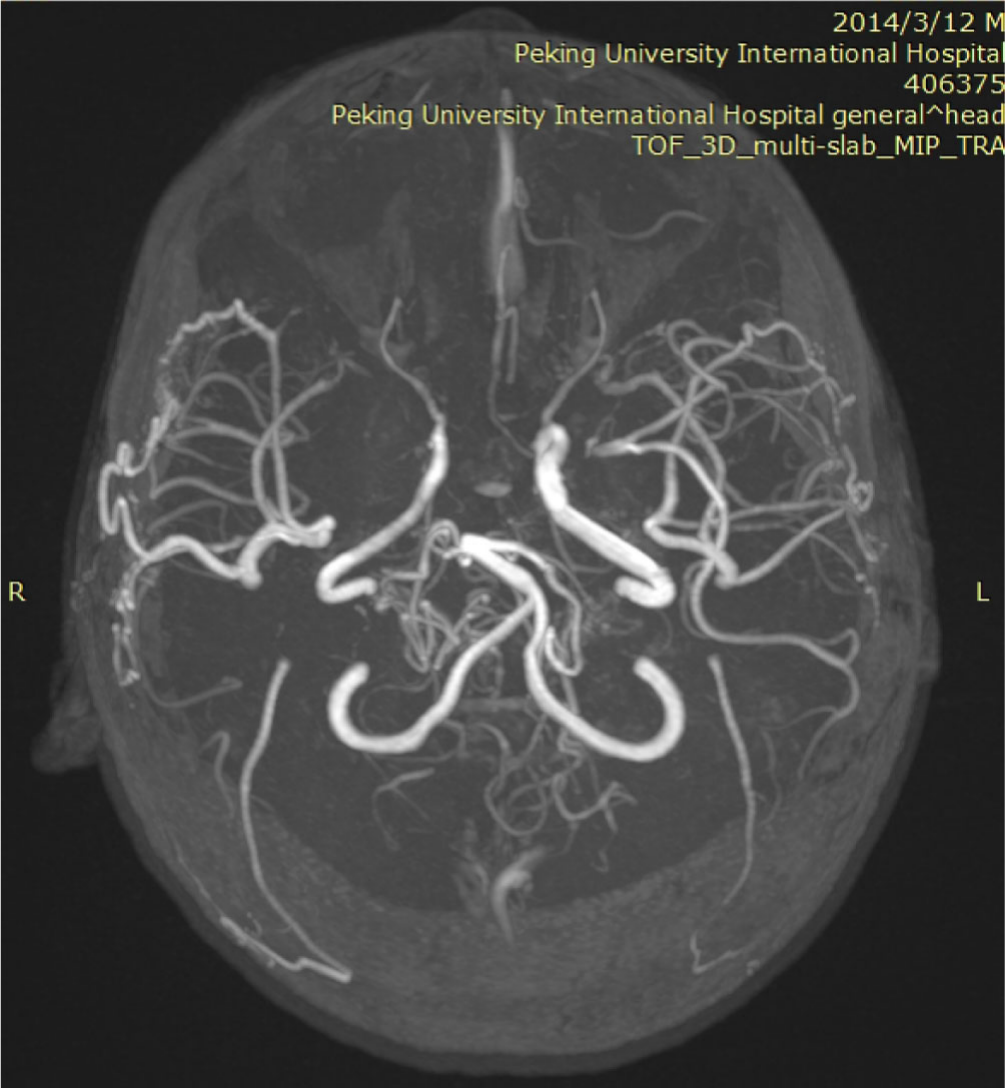
MRA: abundant neovascularization is evident in the bilateral frontal and temporal lobe regions.

During the maintenance phase of treatment, the patient underwent staged bilateral indirect revascularization procedures - encephaloduroarteriosynangiosis (EDAS) at the 53rd and 67th weeks, respectively. MRA performed approximately six months after surgery revealed abundant, tortuous neovascularization in the bilateral frontal and temporal lobes, confirming the successful establishment of collateral circulation (**Figure 3**). To date, the patient remains in complete hematological remission and is free of neurological complications or sequelae.

## 3 Discussion

Moyamoya disease, a rare idiopathic cerebrovascular disorder, is characterized by progressive intracranial arterial occlusion that impairs cerebral perfusion and markedly elevates the risk of ischemic or hemorrhagic stroke. Chemotherapy remains the cornerstone of treatment for B-ALL; however, chemotherapeutic agents induce varying degrees of neurotoxicity, which is one of the most common adverse reactions during treatment. These neurotoxic effects may involve the central nervous system (CNS) and the peripheral nervous system (PNS), with asparaginase and vinca alkaloids being particularly prominent (6). Given that B-ALL typically requires high-intensity chemotherapy regimens to achieve remission, the neurotoxic potential of these drugs may trigger or exacerbate neurological complications in patients.

Moyamoya disease patients face an elevated risk of hypoperfusive cerebral infarction owing to cerebrovascular stenosis and hemodynamic dysfunction. Additionally, the abnormally proliferated collateral vessels characteristic of Moyamoya disease are fragile and prone to rupture, increasing susceptibility to intracranial hemorrhage (1). Moreover, the chemotherapy process may also damage coagulation function, potentially further exacerbating the “thrombosis-hemorrhage” paradox by affecting coagulation function, damaging vascular endothelium, or altering platelet function (9). The interplay of these factors complicates the development and implementation of effective and safe treatment strategies for patients concurrently suffering from Moyamoya disease and B-ALL.

Asparaginase is a key component in the treatment strategy for acute leukemia. While asparaginase inhibits leukemic cell proliferation by depleting circulating asparagine, normal endothelial cells rely on exogenous asparagine for protein synthesis—and antithrombin (AT) production is highly dependent on this amino acid. Consequently, asparaginase therapy may impair endothelial function and reduce AT synthesis, leading to coagulation abnormalities and an increased risk of thrombosis. Meanwhile, as an immunogenic protein, L-asparaginase has the potential to induce hypersensitivity reactions (HSR) in certain patients during therapy, potentially increasing thrombotic risk (10, 11). Several studies have demonstrated that compared with asparaginase preparations derived from *E. coli* strains, Erwinia asparaginase is associated with a lower incidence of neurotoxicity, as well as reduced risks of coagulopathy and allergic reactions (10, 12–14). Furthermore, vindesine has been shown to have lower neurotoxic potential than vincristine (15), making it a potentially preferable option for minimizing neurotoxic burden during chemotherapy.

Additionally, balancing thromboprophylaxis with close monitoring for bleeding is critical during treatment. For ALL patients with concomitant Moyamoya disease, a dynamic assessment of thrombotic and hemorrhagic risks—integrating the pathophysiological features of Moyamoya disease, pharmacological profiles of chemotherapeutic agents, and individual patient factors—is key to guiding clinical decision-making. During high thrombotic risk periods (e.g., significant cerebrovascular stenosis or active pegaspargase therapy), proactive antithrombotic strategies are warranted; conversely, in cases of moderate-to-low thrombotic risk or elevated bleeding risk, antithrombotic intensity should be reduced or paused. Individualized risk stratification and serial monitoring can minimize ischemic stroke and hemorrhage while preserving effective anti-leukemic control—improving long-term outcomes.

Currently, no targeted pharmacotherapy exists for Moyamoya disease. For patients with ischemic symptoms, antiplatelet therapy may be considered. The main treatment methods are intracranial and extracranial vascular reconstruction surgeries, aiming to improve cerebral perfusion in ischemic areas and alleviate neurological dysfunction caused by cerebral ischemia (1, 2, 16, 17).For patients with symptomatic ischemic Moyamoya disease, surgical intervention is recommended to reduce the risk of recurrent ischemic events. In principle, patients with surgical indications should undergo surgical treatment as early as possible, particularly children. In this child, recurrent limb weakness and acute ischemic foci during chemotherapy signaled high progression risk. However, considering the need to complete the subsequent treatment stage of ALL to effectively control the leukemia, a multidisciplinary team (MDT) decided to delay surgery and prioritize re-induction therapy. Factors included acceptable current disease control, slow progression, and minimal impact of short-term surgery delay on prognosis; additionally, interrupting consolidation could raise recurrence risk, and escalating therapy post-recurrence might worsen neurotoxicity. This child successfully underwent vascular reconstruction surgery after completing re-induction therapy, demonstrating the feasibility of a sequential strategy: control leukemia with chemotherapy first, then address Moyamoya disease surgically. Based on the clinical experience of this pediatric patient, we propose that the selection of surgical timing should prioritize the tumor control status. Surgical intervention is recommended when leukemia has achieved complete remission (CR), minimal residual disease (MRD) is undetectable, and the risk of recurrence is low. The optimal time window should avoid periods of high-intensity chemotherapy and preferably be scheduled during the late consolidation phase or maintenance therapy. Furthermore, patients should be in stable general condition, with no evidence of active infection, stable coagulation function, and the ability to tolerate general anesthesia and surgical trauma. However, this strategy requires strict assessment of neurological function and tumor burden: if neurological deterioration occurs during chemotherapy, surgery should be prioritized to preserve brain function, and the chemotherapy regimen should be adjusted accordingly (e.g., dose reduction, cycle extension).

## 4 Conclusion

Although cases of B-ALL coexisting with Moyamoya disease are exceptionally rare, their management presents considerable clinical complexities. While strict adherence to standardized chemotherapy protocols for ALL remains paramount, paramount importance must be accorded to mitigating neurovascular complications associated with moyamoya disease. This mandates a judicious equilibrium between effective anti-leukemic therapy and cerebral vascular protection. Particular attention should be directed toward neurotoxic chemotherapeutics, with therapeutic regimens tailored to minimize risks of central and peripheral nervous system injury through dose optimization and treatment duration adjustments. Concurrently, individualized antithrombotic strategies—including anticoagulation or antiplatelet therapy—require risk-benefit assessments to mitigate ischemic events while avoiding hemorrhagic complications. Multidisciplinary collaboration is essential for developing sequential treatment paradigms that integrate tumor burden, neurological status, neuroimaging dynamics, and pharmacological tolerance. Accumulating robust clinical evidence remains critical to refining evidence-based management frameworks for these complex pediatric patients.

## Data Availability

The original contributions presented in the study are included in the article/supplementary material. Further inquiries can be directed to the corresponding author.
